# The role of pornography in the sex life of young adults-a cross-sectional cohort study on female and male German medical students

**DOI:** 10.1186/s12889-022-13699-4

**Published:** 2022-07-04

**Authors:** Matthias Jahnen, Leopold Zeng, Martina Kron, Valentin H. Meissner, Alexander Korte, Stefan Schiele, Helga Schulwitz, Andreas Dinkel, Jürgen E. Gschwend, Kathleen Herkommer

**Affiliations:** 1 Department of Urology, Klinikum rechts der Isar, School of Medicine, Technical University of Munich, Ismaninger Str. 22, 81675 Munich, Germany; 2 Institute of Epidemiology and Medical Biometry, University of Ulm, Helmholtzstr. 22, 89081 Ulm, Germany; 3 Department of Child and Adolescent Psychiatry, Psychosomatics and Psychotherapy, Ludwig-Maximilians-University Munich, Nußbaumstr. 5a, 80336 Munich, Germany; 4 Department of Psychosomatic Medicine and Psychotherapy, Klinikum rechts der Isar, School of Medicine, Technical University of Munich, Langerstr.3, 81675 Munich, Germany

**Keywords:** Pornography, Social media use, Sexual development, Sexual behavior, Young adults, Gender differences

## Abstract

**Background:**

Due to easy online accessibility of pornography its consumption is popular among adolescents and young adults. Considering recently developed frameworks on the effects of sexual media, we assessed how increased consumption of pornography is associated with the experience of certain aspects of offline and online sexual activity in German medical students.

**Methods:**

Between April 2018 and March 2020 medical students from the Technical University of Munich in Germany were anonymously surveyed with regards to their sexual behavior, consumption of pornography, and use of social media. 468 students (female: *n* = 293; male: *n* = 175) were included in the current analysis. Data was analyzed using simple and multiple Poisson regression analysis.

**Results:**

Only 7.3% of female students but the majority of male students (79.1%) consumed pornography more than 4 times in the last 4 weeks. Female and male students who reported to be inspired by pornography (female: 52.0%, male: 74.6%) and who have enjoyed the experience of anal intercourse in their life (female: 17.1%, male: 32.2%) consumed pornography more frequently. Female students who have experienced a “threesome” (9.0%), have sent erotic pictures of themselves (33.5%), or use social media in their dating life (27.6%) consumed pornography more frequently. Male students who did not experience a sexual transmitted disease (82.9%) and did not cheat on their partner (68.0%) consumed pornography more frequently (results of multiple Poisson regression analysis; all *p* < 0.05).

**Conclusions:**

Many students consider pornography as inspiration for their sex life and frequent consumption of pornography seems to be associated with gender specific characteristics congruent with short-term sexual quality. The desire of adolescents and young adults for practical information about sexual intercourse should be addressed openly and a proper understanding of the sexuality shown in pornography should be taught.

## Background

Aided by the easy online accessibility of more than 10,000 Terabytes of pornographic content, frequent consumption of internet pornography, particularly among young adolescents and adults, has become increasingly popular [1, 2]. Pornhub, one of the most popular websites for pornographic material is visited over 40 Billion times annually, and studies report that up to 90% of adults below the age of 35 have consumed pornography in the last year [2–5].

The displayed sexuality (sexual scripts) in pornography is in general considered to focus on short-term sexual quality, in which sexuality is considered more casual and disconnected from long-term relationship commitments [6, 7]. Further, these sexual scripts are also in most cases incongruent with long-term sexual quality, which is driven by traditional, long-term, monogamous relationship values. Therefore, it has been suggested in previous studies that regular consumption of pornography might be associated with negative effects regarding long-term sexual satisfaction, relationship satisfaction, and sexual health [8, 9]. For clarity, what we are referring to as ‘short-term sexual quality’ is no way less valuable than ‘long-term sexual quality’, these are different, but equally valuable concepts of sexuality.

Research on how the consumption of pornography impacts sexual health and sexual behavior acted out in one’s real life, has come up with mixed results. Studies have on the one hand shown associations between the consumption of pornography with sexual behavior not focused on long-term relationship quality and congruent with more adventures, casual sexuality (e.g. frequently changing sexual partners, one night stands). On the other hand studies have also shown associations with heightened long-term intimate sexual quality by e.g. improving sexual communication and experimentation [10–14]. Further, whether these potential influences on sexual behavior lead to measurable changes in sexual satisfaction or a negative impact on sexual health (e.g. contraction of a sexually transmitted disease (STD)) in the general public remains unclear. To nuance the implications drawn from these studies, several frameworks have been established in recent years in conceptualizing in which way the consumption of pornography may affect sexual behavior, quality, and health [6, 7, 12]. These frameworks propose that consumption of sexual content has the potential to lead to the adaptation of the displayed sexual scripts in one’s sexual behavior. However, the adaptation of these scripts is highly moderated by several factors such as exclusivity (pornography is the only source of role modeling sexual behavior), formativeness (young age of exposure), resonance (congruence with real-life sexual experience), and reinforcement (positive feedback from enacting particular sexual scripts) [6, 7]. Whether the adaptation of sexual scripts depicted in pornography may lead to a decrease of long-term sexual quality or promotion of certain sexual behavior is further influenced by contextual factors such as use patterns and moral paradigms [6, 12]. Under consideration of these frameworks, we assessed the viewing patterns of sexual media and its association with sexual behavior in a highly educated sample of German medical students. We hypothesized that due to easy access, the liberal social structure and an in general high cultural openness to sexuality the consumption of pornography as well as the frequency of consumption will be high in our sample. Further, we estimated that due to the high educational background of our sample associations between consumption of pornography – defined as explicit sexual media depicting specific sexual acts – and sexual behavior measurable but won’t affect overall sexual satisfaction and sexual health. To create an overview in which way the surveyed students act out their sexuality, a broad set of items focusing on previously proposed factors associated with short-term sexual quality (online and offline sexual openness and sexual technique) were assessed. Thirdly, we analyzed whether gender-specific characteristics that are associated with the consumption of pornography and certain sexual behavior can be identified.

## Material and methods

### Study sample

Between April 2018 and March 2020, fourth- and fifth-year medical students from the Technical University of Munich in Germany participated in an anonymous survey regarding their sexual behavior. Self-reporting questionnaires were handed out during a urology seminar, and a private space for the students to fill out the questionnaire was provided. The questionnaire version for female and male students was mostly identical except for a few gender specific questions. Students were given the choice to pick the questionnaire for the gender they identify with. Students with a non-binary gender identity were given the choice to either fill out the questionnaire they are most comfortable in answering or to decline study participation (*n* = 1). All students who provided information on their consumption of pornography were included into the analysis. In total, 479 students were asked to participate, 6 declined (response rate: 98.8%). 468 students (female: *n* = 293; male: *n* = 175) who completed the questionnaire with regards to their consumption of pornography were included.

This study was approved by the ethics commission of the Technical University of Munich.

### Sociodemographic characteristics and self-perception

Students were asked about their age, height, weight, whether they were in a stable intimate relationship and the duration of their current relationship. We assessed the following aspects of self-perception: satisfaction with one’s physical appearance (no/yes (Scale from 1 (extremely unsatisfied) to 10 (extremely satisfied)); responses below 6 were considered as “no”)); subjective perception of penile length (normal/not normal (only male students)).

### Consumption of pornography

To reproducibly assess pornographic consumption, pornography in this study was defined as media depicting genitals or sexual intercourse. This kind of media has previously also been defined as sexually explicit media [4, 6, 15].

We assessed the following aspects of the student’s consumption of pornography: Lifetime consumption of pornographic material (no/yes); frequency of consumption of pornography (total number of times in the last 4 weeks); masturbation while consuming pornography (never/sometimes/most times/always); media of consumption of pornography (movie, pictures, books/written); kind of pornography (Fig. 2; for particular content such as BDSM, gay, lesbian or violent definition was given for better understanding); inspired by pornography (no(`no, not at all´)/maybe(`I think there might be an inspiration, but I cannot estimate the impact of pornography on my sex life´)/yes(`Yes, I feel very inspired’; `Yes, I feel moderately inspired´, `Yes I feel somewhat inspired´)); consumption of pornography with someone else (partner, friends, strangers (no/yes)); sending of pornographic material via digital devices (no/yes).

### Sexual behavior, sexual satisfaction, sexual health

We assessed the following general aspects of the student’s sex life with separate single items: sexual identity (heterosexual/mostly heterosexual/bisexual/mostly homosexual/homosexual); partnered sexual activity (total over the last 4 weeks); masturbation (total over the last 4 weeks); number of sexual partners in life; satisfaction with one’s sex life (no/yes); sexually transmitted disease (STD) in life (no/yes).

#### Sexual openness

Use of social media for dating or sex life (no/yes); sending of erotic pictures of one’s self to one’s partner or to strangers (no/yes). Sexual intercourse with multiple partners at the same time (no/yes); cheating on one’s partner in life (no/yes);

#### Sexual technique

Experience of vaginal, oral and anal intercourse in life (no/yes); enjoyment of vaginal, oral and anal intercourse in life (no […] intercourse/never, only a few times/ at least some times); condom use during vaginal (last 4 weeks), oral (last 4 weeks) and anal intercourse (last year) (no […] intercourse/never, a few times, sometimes/at least most times).

### Statistical analysis

Descriptive statistics were calculated for all study variables. Chi-square and Wilcoxon rank-sum tests were applied for analyzing differences between male and female students. Multiple Poisson regression analysis were used for female and male students separately to identify variables associated with the frequency of pornographic consumption in the last 4 weeks. Backward elimination (using a selection level of 5%) was applied for variable selection. Relative risks with 95% confidence interval were calculated in the final model. All statistical tests are exploratory in nature. All analyses were performed using SAS (Version 9.4).

## Results

### Students characteristics

Median age of the surveyed students was 24.0 years. Two thirds of the students were in a relationship with a median duration of 1.1 year. Most students (female: 89.8%, male: 93.1%) were satisfied with their physical appearance (Table 1).

**Table 1:** Characteristics of the study sample (*N* = 468)

	All (***N*** = 468)		Female students (***n*** = 293)		Male students (***n*** = 175)	
Characteristics	n	%	n	%	n	%
**Sociodemographic and lifestyle factors**						
**Age (*years*) Med (1st-3rdQ)**	24.0 (23.0–26.0)		24.0 (23.0–25.9)		24.0 (23.0–26.0)	
**BMI (*kg/m***^***2***^) M (SD) *	21.9 (3.0)		21.0 (2.8)		23.4 (2.7)	
**Current partnership**						
**No**	154	33.0	91	31.1	63	36.2
**Yes**	313	67.0	202	68.9	111	63.8
**Partnership duration (years) Med (1st-3rdQ)**	1.1 (0–3.6)		1.1 (0–3.5)		1.0 (0–3.9)	
**Social media use and self-perception**						
**Social media use for dating**						
**No**	335	71.6	212	72.4	123	70.3
**Yes**	133	28.4	81	27.6	52	29.7
Social media use for sex life *						
**No**	362	77.9	236	81.4	126	72.0
**Yes**	103	22.1	54	18.6	49	28.0
Sending erotic pictures of one’s self to partner*						
**No**	332	72.2	191	66.5	141	81.5
**Yes**	128	27.8	96	33.5	32	18.5
Sending erotic pictures of one’s self to strangers*						
**No**	452	97.4	285	98.6	167	95.4
**Yes**	12	2.6	4	1.4	8	4.6
**Satisfaction with physical appearance**						
**No**	42	9.0	30	10.2	12	6.9
**Yes**	426	91.0	263	89.8	163	93.1
**Subjective penile length**						
**Normal**	–	–	–	–	144	82.3
**Not normal**	–	–	–	–	31	17.1
**Sex Life**						
Sexual identity *						
**Heterosexual**	369	79.3	225	77.3	144	82.8
**Mostly heterosexual**	73	2.4	55	18.9	18	10.3
**Bisexual**	11	2.6	8	2.8	3	1.7
**Mostly homosexual/homosexual**	12	15.7	3	1.0	9	5.2
**Satisfied with sex life**						
**No**	108	23.6	63	22.1	45	26.0
**Yes**	350	76.4	222	77.9	128	74.0
**Partnered sexual activity (total over last 4 weeks) Med (1st-3rdQ)**	4.0 (0.0–10.0)		4.0 (0.0–10.0)		4.0 (0.0–9.5)	
**Masturbation (total over last 4 weeks)** Med (1st-3rdQ) *	5.0 (2.0–10.0)		3.0 (1.0–5.0)		10.0 (6.0–20.0)	
**Number of sexual partners (in life) Med (IQR)**	5.0 (2.0–8.0)		4.0 (2.0–8.0)		5.0 (2.0–8.0)	
**Vaginal intercourse (in life)**						
**No**	40	8.6	25	8.6	15	8.6
**Yes**	424	91.4	265	91.4	159	91.4
**Enjoyment of vaginal intercourse (in life)**						
**No vaginal intercourse**	35	7.6	22	7.6	13	7.5
**Never/few times**	7	1.5	5	1.7	2	1.2
**At least sometimes**	420	90.9	262	90.7	158	91.3
**Use of Condoms during vaginal intercourse (last 4 weeks)**						
**No vaginal intercourse**	36	7.9	22	7.7	14	8.2
**Never/a few times/sometimes**	218	47.8	140	48.0	78	45.9
**At least most times**	202	44.3	124	43.4	78	45.9
**Oral intercourse (in life)**						
**No**	26	5.6	18	6.2	8	4.6
**Yes**	440	94.4	274	93.8	166	95.4
**Enjoyment of oral intercourse (in life)**						
**No oral intercourse**	25	5.4	18	6.2	7	4.1
**Never/few times**	23	5.0	15	5.2	8	4.6
**At least sometimes**	415	89.6	257	88.6	158	91.3
**Use of Condoms during oral intercourse (last 4 weeks)**						
**No oral intercourse**	28	6.2	20	7.0	8	4.7
**Never/a few times/sometimes**	411	90.1	258	89.9	153	90.6
**At least most times**	17	3.7	9	3.1	8	4.7
**Anal intercourse (in life)**						
**No**	309	66.3	197	67.5	112	64.4
**Yes**	157	33.7	95	32.5	62	35.6
**Enjoyment of anal intercourse (in life)**						
**No anal intercourse**	308	66.0	197	67.2	111	63.8
**Never/few times**	53	11.3	46	15.7	7	4.0
**At least sometimes**	106	22.7	50	17.1	56	32.2
**Use of Condoms during anal intercourse (last year)**						
**No anal intercourse**	308	66.3	197	67.2	111	64.5
**Never/a few times/sometimes**	88	18.9	51	17.4	37	21.5
**At least most times**	69	14.8	45	15.4	24	14.0
Sexual intercourse with multiple partners at the same time (in life) *						
**No**	403	87.6	262	91.0	141	82.0
**Yes**	57	12.4	26	9.0	31	18.0
**Cheating on partner (in life)**						
**No**	332	70.9	213	72.7	119	68.0
**Yes**	30	6.4	14	4.8	16	9.1
**Sexual transmitted disease (in life)**						
**No**	395	15.6	250	85.3	145	82.9
**Yes**	73	84.4	43	14.7	30	17.1

Notes: * indicating statistically significant (*p* < 0.05) differences in the comparison of female and male students; *M* mean, *SD* standard deviation, *Med* median, *1st-3rdQ* 1st to 3rd quartile, *BMI* body mass index; missing data per characteristic ranged from 0 to 106

### Consumption of pornography

**Fig. 1:**
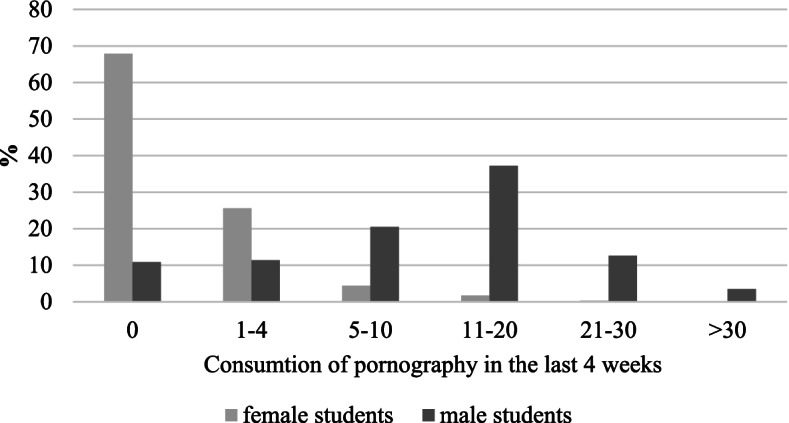
Total number of times female and male students consumed pornography in the last 4 weeks

Only 7.3% of female students but the majority of male students (79.1%) consumed pornography more than 4 times in the last 4 weeks. Lifetime consumption of pornographic material (female: 71.7% vs. male: 96.6%) was also higher in male than female students (Fig. 1 and Table 2) (*p* < 0.01).

**Table 2:** Details on the consumption of pornographic material of the study sample

Characteristics	All (***n*** = 468)		Female students (***n*** = 293)		Male students (***n*** = 175)	
	n	%	n	%	n	%
**Consumption of pornographic material (in life)**						
**No**	89	19.0	83	28.3	6	3.4
**Yes**	379	81.0	210	71.7	169	96.6
**Porn consumption (total in last 4 weeks)** Med (1st-3rdQ) *	–		0 (0–2)		12 (6–20)	
Masturbation while consuming pornography *						
**Never**	103	23.2	101	37.2	2	1.2
**Sometimes**	61	13.7	52	19.2	9	5.2
**Most times**	110	24.8	50	18.5	60	34.7
**Always**	170	38.3	68	25.1	102	58.9
Consumption of pornography as movie*						
**No**	110	29.0	81	38.6	29	17.2
**Yes**	269	71.0	129	61.43	140	82.8
Consumption of pornography as pictures*						
**No**	291	76.8	176	83.8	115	68.1
**Yes**	88	23.2	34	16.2	54	31.9
Consumption of pornography as books/written*						
**No**	321	84.7	161	76.7	160	94.7
**Yes**	58	15.3	49	23.3	9	5.3
Inspired by pornography*						
**No**	78	17.6	64	23.8	14	8.1
**Maybe**	95	21.5	65	24.2	30	17.3
**Yes**	269	60.9	140	52.0	129	74.6
Porn consumption with someone else*						
**No**	258	68.1	126	60.0	132	78.1
**Yes**	121	31.9	84	40.0	37	21.9
Porn consumption with partner*						
**No**	278	73.4	139	66.2	139	82.3
**Yes**	101	26.6	71	33.8	30	17.7
**Porn consumption with friends**						
**No**	346	91.2	188	89.5	158	93.5
**Yes**	33	8.7	22	10.5	11	6.5
**Porn consumption with strangers**						
**No**	378	99.7	210	100.0	168	99.4
**Yes**	1	0.3	0	0.0	1	0.6
Sending of pornographic material*						
**No**	437	94.0	277	95.5	160	91.4
**Yes**	28	6.0	13	4.5	15	8.6

Notes: * indicating statistically significant (*p* < 0.05) differences in the comparison of female and male students; *Med* median, *1st-3rdQ* 1st to 3rd quartile; missing data per characteristic ranged from 0 to 24

**Fig. 2:**
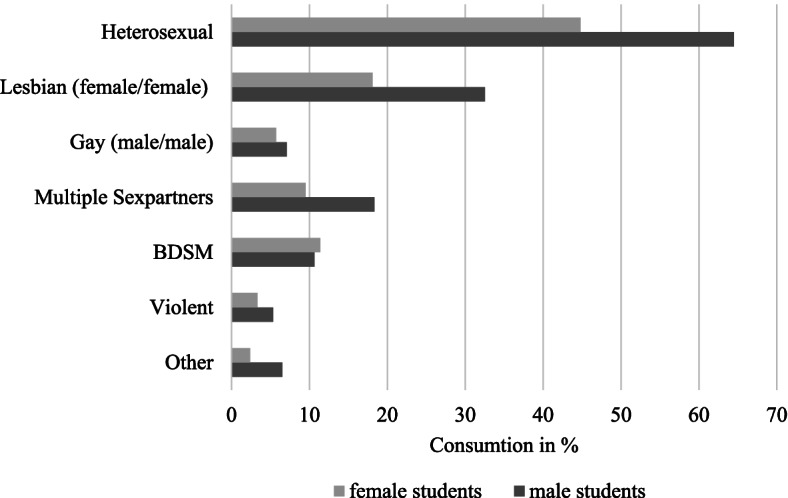
Percentage of female and male students that consume certain kinds of pornography (Notes: BDSM Bondage, Discipline, Dominance and Submission, Sadism and Masochism)

Female students reported less often to masturbate `at least most times´ while consuming pornography compared to male students (female: 43.6% vs. male: 93.6%). Most pornography was consumed as movie (71.0%), and female students reported more often to consume pornography in written form (female: 23.3% vs. male: 5.3%). Less female students reported to be inspired by pornography than male students (female: 52.0% vs. male: 74.6%). 33.8% of female students reported to consume pornography with their partner compared to 17.7% of male students. (all *p* < 0.05) (Table 2) Details on the kind of pornography consumed are displayed in Fig. 2.

### Sexual behavior, sexual scripts, and sexual health

Most students identified themselves as heterosexual (female: 77.3%; male: 82.8%). The sex life of female and male students did not differ with regards to frequency of partnered sexual activity and number of lifetime sexual partners. However, female students masturbated less often than male students in the last 4 weeks (female: Med = 3.0 (IQR 1.0–5.0) vs. male: Med = 10.0 (6.0–20.0)) (*p* < 0.05). Less than 20% of students experienced a STD in their life (female: 14.7%; male 17.1%).

Less than a third of students used social media/dating software as part of their dating life (female: 27.6%; male: 29.7%) or sex life (female: 18.6%; male: 28.0%). 33.5% of female students and 18.5% of male students reported to have sent erotic pictures of themselves to their partners (*p* < 0.05). More male students reported to have sent erotic pictures of themselves to strangers (female: 1.4% vs male: 4.6%) (*p* < 0.05). 9% of female students and 18.0% of male students were part of sexual intercourse with multiple partners at the same time (*p* < 0.05). Less than 10% of students reported to have cheated on their partner in their life (female: 4.8%; male: 9.1%).

The sex life of female and male students did not differ with regards to experience and enjoyment of vaginal and oral intercourse, but less female students enjoyed anal intercourse than male students (female: 17.1% vs. male: 32.2%) (*p* < 0.05) (Table 1).

### Characteristics associated with more frequent consumption of pornography in the last 4 weeks

Multiple Poisson regression analysis with backward elimination (Table 3) was used to identify variables associated with the amount of consumption of pornography in the last 4 weeks for female and male students.

**Table 3:** Factors associated with the consumption of pornography in female and male students - results of multiple Poisson regression with backward elimination

Characteristics	Female students (***n*** = 293)		Male students (***n*** = 175)	
	*RR [95% CI]*	*p*	*RR [95% CI]*	*p*
**Age** ***(continuous)***	0.92 [0.87–0.96]	< 0.001	–	–
**BMI** ***(continuous)***	1.12 [1.07–1.16]	< 0.001	–	–
**Satisfaction with physical appearance** ***(ref. no)***				
**Yes**	4.13 [2.11–8.07]	< 0.001	–	–
**Social media use for dating** ***(ref. no)***				
**Yes**	1.27 [1.01–1.59]	0.040	–	–
**Sending erotic pictures of one’s self** ***(ref. no)***				
**Yes**	1.35 [1.07–1.70]	0.012	1.15 [0.97–1.28]	0.064
**Inspired by pornography** ***(ref. no)***		< 0.001		< 0.001
** Maybe**	1.77 [1.15–2.70]		2.36 [1.71–3.27]	
** Yes**	2.67 [1.82–3.94]		2.69 [1.97–3.68]	
**Sexual orientation** ***(ref. heterosexual)***		< 0.001		0.117
** Mostly heterosexual**	1.55 [1.23–1.97]		0.99 [0.86–1.13]	
** Bisexual**	2.22 [1.43–3.45]		0.59 [0.40–0.87]	
**Mostly homosexual/homosexual**	2.28 [1.07–4.87]		0.86 [0.71–1.04]	
**Partnered sexual activity (total over last 4 weeks)** ***(continuous)***	0.98 [0.96–0.99]	0.009	–	–
**Masturbation (total over last 4 weeks)** ***(continuous)***	1.06 [1.04–1.07]	< 0.001	1.04 [1.03–1.05]	< 0.001
**Enjoyment of anal intercourse (in life)** ***(ref. at least sometimes)***		< 0.001		< 0.001
** No anal intercourse**	0.57 [0.44–0.74]		0.77 [0.70–0.85]	
** Never/few times**	0.89 [0.66–1.21]		0.69 [0.55–0.87]	
**Sexual intercourse with multiple partners at the same time (in life)** ***(ref. no)***				
**Yes**	1.55 [1.18–2.03]	0.002	–	–
**Cheating on partner (in life)** ***(ref. no)***				0.005
** Yes**	–	–	0.81 [0.70–0.94]	
** No answer**	–	–	1.10 [0.99–1.22]	
**Sexual transmitted disease (in life)** ***(ref.no)***				
**Yes**	1.60 [1.23–2.09]	< 0.001	0.77 [0.68–0.88]	< 0.001

Notes: - indicating the removal of the factor from the final statistical model; *RR* Relative Risk, *CI* confidence interval, *ref.* reference, *BMI* body mass index

For female and male students the following associations were found:

Female and male students who stated to be inspired by pornography consumed 2.76 [1.88–4.05] and 2.81 [2.06–3.84] times more often pornography than students who were not inspired by pornography. Female and male students who masturbated more often consumed more often pornography (1.07 [1.05–1.09] and 1.04 [1.03–1.05], respectively). Female students who did not have a strictly heterosexual orientation consumed pornography more often than strictly heterosexual female students (mostly heterosexual 1.50 [1.19–1.90], bisexual 2.12 [1.36–3.30], mostly homosexual/homosexual 2.01 [0.94–4.30]). Male students with a bisexual orientation consumed 0.59 [0.40–0.87] times less often pornography while male students with a mostly homosexual/homosexual orientation consumed 1.67 [1.09–2.57] times more often pornography than strictly heterosexual male students. Female and male students who stated to enjoy anal intercourse at least sometimes consumed 1.72 [1.36–2.26] and 1.34 [1.18–1.43] times more often pornography compared to students who did not have anal intercourse (Table 3).

Exclusively for female students the following associations were found:

Female students who used social media for dating or had sexual intercourse with multiple partners at the same time consumed 1.26 [1.01–1.58] and 1.44 [1.09–1.92] times more often pornography compared to female students who did not report such behavior. Female students who have sent erotic pictures of themselves to their partners consumed 1.32 [1.05–1.68] times more often pornography compared to students who did not report such behavior. Female students who were satisfied with their physical appearance watched 3.18 [1.60–6.33] times more often pornography than the ones who were not. Female students who reported the contraction of a STD in their life watched 1.60 [1.23–2.09] times more often pornography than the ones who did not report the contraction of a STD (Table 3).

Exclusively for male students the following associations were found in multiple regression analysis:

Male students who have had experienced anal intercourse but did not enjoy it consumed 0.72 [0.57–0.90] times less often pornography compared to male students who stated to enjoy anal intercourse at least sometimes. Male students who reported to have cheated on a partner watched 0.82 [0.70–0.95] times less often pornography compared to male students who did not (Table 3).

## Discussion

The consumption of pornography is popular due to the fast experience of sexual arousal and it is increasingly common because of its easy access through the internet [8, 16]. As expected, in this analysis on German medical students with a mean age of 24.0 years the majority of male students (96.6%) and female students (71.7%) reported that they have consumed pornographic material in the past. Further, 79.1% of male students reported consumption of pornographic material in the last 4 weeks and more than a third indicated consumption of pornographic material daily or every other day. Also, a third of female students reported consumption of pornographic material in the last 4 weeks but with an on average lower frequency than male students. Moreover, two-thirds of the students considered pornography to have inspired their own sex life in some way underlining the central role of pornography as a source of information in the sex life of young adults. These findings follow previous research, showing that consumption of pornographic material is high especially among young male adults who, in general, consume significantly more pornographic material than their female counterparts [17–19]. Nevertheless, the frequency of regular consumption of pornography in this analysis is 20–30% higher than reported in most other studies on the matter [1, 8]. Comparison with data from Western countries with similar sociocultural liberties towards sexuality such as Denmark and the Netherlands also shows equal patterns in the consumption of pornography among young adults [3, 18]. Still, an increase in the consumption of pornography due to further digitalization and improvement of mobile internet access in recent years is expected when comparing this data with data from the early 2000s. This assumption is supported by the finding that the most common source of pornography for female and male students were videos which are usually distributed via the internet. Analysis of watched content revealed, that in most cases the consumed content is the depiction of heterosexual, lesbian (female on female), or gay (male on male) intercourse. Only a minority of 10% reported having ever consumed paraphilic sexual content depicting bondage, domination, or violence. These data are in accordance with previous studies also showing that the consumption of paraphilic sexual media remains a niche despite its easy accessibility, contradicting the previously suggested fear that frequent, unrestricted access to pornographic material might trigger a continuous normalization of paraphilic sexuality [7, 20].

Studies investigating the influence of the consumption of pornography on sexual behavior suggest that the effects of pornography on real-life sexual scripts and sexual quality are individually influenced by moderators and contextual factors [7, 12]. We hypothesized that in our sample of on average 24-year-old German medical students, due to their liberal and highly educated background, associations with particular sexual behavior might be measurable but without a significant impact on overall satisfaction or sexual health. To test this theory, we deployed multivariate Poisson regression analysis controlling for basic sociodemographic and sexual characteristics to identify associations between the frequency of pornography consumption and sexual behavior patterns. We focused primarily on sexual behavior patterns that highlight sexual technique and sexual openness. In this regard, results from this analysis revealed that female and male students who have enjoyed the experience of anal intercourse in their life consume pornography more often than students who have had not practiced anal intercourse. Anal intercourse is commonly practiced in pornography and portrayed as enjoyable. Previous studies have shown similar associations between frequent consumption of pornography and the experience of anal intercourse [3, 21]. Our data add to these previous findings that not only the application of sexual technique but also the enjoyment of those techniques seem to be associated with frequent consumption of pornography underlining the suggested role of resonance as a positive feedback loop in the above mention framework. Another reoccurring theme in pornography is the display of sexual intercourse with multiple partners as a pleasurable experience. The results of this analysis show that female students who have experienced sexual intercourse with multiple partners at the same time also consume pornography more often. This suggests an association between consumption of pornography and sexual openness to extraordinary sexual experiences. Aspects of sexual openness are also expressed in the interaction and communication with potential partners for relationships, hook-ups, or online sexual encounters. Especially in recent years, social media and dating software are increasingly popular in providing scarcely regulated opportunities to connect with a vast pool of potential new partners. This gives primarily sexually open individuals with low sexual anxiety the opportunity to connect with like-minded individuals in the pursuit of sexual interaction and satisfaction [22]. Our results show associations with expressed sexual openness and low sexual anxiety during the use of modern media with frequent consumption of pornography. Female students who reported to have sent erotic pictures of themselves also consumed more often pornography compared to female students who did not. Moreover, female students who used social media and mobile dating apps in their dating life reported higher consumption of pornography. These results indicate that adults who are more accustomed to a visual display of sexuality might also be more comfortable in sharing similar images of themselves. Being used to experiencing some part of one’s sex life online through the consumption of pornography, seems also associated with openness to the use of mobile dating apps.

Interestingly, results of multivariate Poisson regression analysis revealed that a higher frequency of pornographic consumption was negatively associated with being unfaithful among male students. Cheating on one’s partner is often the result of unmet intimate desires in one’s relationship and can be considered as valuing short-term sexual desire over long-term sexual and relationship quality [6].

Taken together our findings on sexual openness and technique show overall congruent results with established frameworks on the relationship between sexual behavior focusing on short-term sexual quality and the consumption of pornography. Remarkably, although the frequency of pornographic consumption was considerably higher among male students, the identified associations between consumption of pornography and sexual behavior attributed with short-term sexual quality were more prominent in female students. These discrepancies might be explained by further contextualizing the consumption of pornography. Factors such as the use patterns and script application in a couple-context can further modulate the potential influence of pornography on sexual behavior [6, 12]. In this analysis, most male adults reported consuming pornography at least once a week, most of the time alone without a partner or a friend. Female students reported significantly less consumption of pornography and more often in the presence of their partner or a friend. Further, our data shows that nearly all male students (93.6%) masturbate `at least most times´ during the consumption of pornography, while such behavior was reported by less than half of the female students (43.6%). These results suggest that the underlying motivation for female and male adults to consume pornography might differ and might therefore impact real-life sexual behavior differently. For young women, consumption of pornography might be more often a source of information or entertainment and part of an openly expressed sexuality to be consumed with friends or a partner and might therefore interact more directly with sexual scripts acted out with real life sexual partners [16]. In male adults, the consumption of pornography seems to be closely related to masturbation and is practiced regardless of other partnered sexual activity and might serve for many young men as an outlet to satisfy short-term sexual drive motivation [14].

It is an ongoing discussion, whether the potential promotion of sexual behavior focusing on short-term sexual quality by the consumption of pornography might lead to long-term sexual dissatisfaction and might have a negative impact on sexual health [8, 23]. We hypothesized that due to the liberal and educated background of our study sample there would not be such a measurable association between overall dissatisfaction with one’s sex life and frequent consumption of pornography. This hypothesis was met as we could not identify an association between sexual dissatisfaction and frequent consumption of pornography in female or male students. This is in accordance with a recent longitudinal study that demonstrates that most consumers of pornography do not experience a decrease in satisfaction with their sex life as they can differentiate pornography from real partnered sexuality and some couples report that consumption of pornography might also add fulfilling aspects to one’s sex life [11, 24, 25].

Moreover, the frequent confrontation with exaggerated display of sexuality in pornography is believed to lead to dissatisfaction with one’s physical appearance and sexual ability when they do not meet the heightened expectations [8, 23]. With regards to body satisfaction, the results of this analysis show that female students who were satisfied with their physical appearance consume 3 times as much pornography than female students who reported to be unsatisfied. Dissatisfaction with one’s physical appearance might restrain young women in engaging in the consumption of pornography, that most often present women in a way that is considered visually pleasing to men, in order to avoid feelings of inadequacy.

With regards to sexual health one leading concern expressed repeatedly is that the consumption of pornography might promote sexual “risk behavior”, that leads to the increased chance of contracting a STD [26]. Pornography is produced in order to display boundless, open sexuality and deliberately illustrates in many cases unprotected sexual intercourse with “strangers” and the switching of sexual positions in extreme ways, such as the frequent changing between vaginal, anal and oral intercourse. Such sexual behavior is often considered as sexual risk behavior as it is associated with the increased likelihood of contracting a STD, which can have lasting health consequences. The rising incidence of STDs among young adults has been of growing concern and it is the obligation of the medical community to promote safe sex in order to prevent further spread of diseases such as Chlamydia, Gonorrhea, and HIV [26]. In our analysis, we aimed to assess the association between more frequent consumption of pornography and the risk of contracting a STD. In this regard results of multiple Poisson regression analysis, factoring in sexual frequency, lifetime sexual partners and a variety of sexual practices, show that female students who have experienced a STD in their life consumed 1.6 times more often pornography compared to female students who have not. These results are in line with our findings on the relationship between sexual openness and the consumption of pornography in women. Women who seem more interested in pornography seem to engage in general in a more adventurous sex life which might also carry an increased risk for contracting STDs. Among male students consumption of pornography was slightly higher in the students without the experience of a STD and no associations between condom use during various sexual acts and pornographic consumption were found. These results indicate that the broad consumption of pornography from young male adults plays a neglectable role in the spread of STDs. Nevertheless, due to their wide spread reach, distributors of pornography should consider increasing the promotion of safer sex and stressing the importance of regular health checkups when engaging in a polygamous, more adventures sexual life style.

Results of this analysis have to be considered with certain limitations. For once, due to the cross-sectional, exploratory nature of our study, the associations identified don’t equal causal connections and have to be further investigated in longitudinal studies. When considering our findings on online and offline sexual behavior, certain personality traits might mediate certain effects. Previous research has pointed out that young adults who are by nature high in openness to sexual experience tend to live an in general more sexually adventures and experienced lifestyle, while also being extraordinarily interested in pornography [3, 27]. Further, the identified associations in this analysis might be also explained by pre-existing sexual interests, that were developed independently from pornography. Adults who are more interested in sexual practices, such as anal intercourse or a threesome, might consume pornography more often as it displays sexuality similar to their sexual scripts and desires [28].

Moreover, this analysis includes only German medical students and might therefore be not representative of the general public and young adults with other socio-cultural backgrounds. Due to the high educational background of our sample, knowledge about sexual health, contraception, and the over-exaggeration of sexual practice in pornography have to be considered higher in our sample than in the general public. Succeeding studies should consider acquiring more information on the first encounter with sexually explicit media (e.g. age at first exposure, voluntary or involuntary exposure), consumption of extreme/violent content, payment for consumption of pornography and how the consumption of pornography might change during the time of emerging adolescents and young adulthood to grasp its impact on sexual development. Our study is limited in this regard as it did not focus on the identification of extreme or pathological behavior patterns and by its cross-sectional design. Further, it has to be noted that limited by the available space of the questionnaires many variables were measured using single items. Although, this might decrease the validity of our measured sociopsychological variables, previous research has shown that using single items with high factor loading can produce highly credible measurements, when longer questionnaires cannot be applied [29, 30]. Lastly, due to the sample size, limited data has been generated on the role of pornography for non-heterosexual, non-binary, diverse individuals in these study. These aspects should be further investigated in future studies. Nevertheless, due to the high participation rate and detailed exploration of many aspects of sexuality this analysis gives valuable insights into the role of pornography in the sex life of young adults.

## Conclusions

To conclude, the results of this analysis show that the consumption of pornographic material is highly common among young German medical students. Many students consider it as an inspiration for their own partnered sex life and associations between frequent consumption of pornography and aspects of sexual behavior congruent with short-term sexual quality were identified without a measurable reduction in overall sexual satisfaction. Although the consumption of pornography was more common among men, associations between the consumption of pornography and sexuality were more evident in women. This might be the result of differences in the underlining motivation for the consumption of pornography. We, therefore, suggest that gender roles should be considered in future studies and frameworks outlining the influence of pornography on sexual behavior and quality. We hypothesized that due to our unique study sample we could assess associations between frequent consumption of pornography and sexual behavior in highly educated young adults with. Therefore, results from this analysis might act as an outline on how the role of pornography might be considered in a liberal, sexually open-minded culture which offers broad sexual health education for adolescents and young adults. Educators and medical professionals need to consider that consumption of pornography has become an elemental part of sexuality among young adults. One way to tackle the existing concerns about pornography is to openly address the desire of adolescents and young adults for practical information about sexuality and to teach that sexuality has many faces and sexual interests can vary widely between individuals. Further, with increasing sexual openness it is important to repeatably and publicly promote the concept of consent, safer sex and gender equality.

## Data Availability

All complementary data that support the findings of this study are available from the corresponding author, M. Jahnen upon reasonable request.

## References

[1] Peter J, Valkenburg PM (2016). Adolescents and pornography: a review of 20 years of research. J Sex Res.

[2] Pornhub. The 2019 Year in Review. 2019. https://www.pornhub.com/insights/2019-year-in-review. Accessed 1 July 2022.

[3] Hald GM, Kuyper L, Adam PCG, de Wit JBF (2013). Does viewing explain doing? Assessing the association between sexually explicit materials use and sexual behaviors in a large sample of Dutch adolescents and young adults. J Sex Med.

[4] Morgan EM (2011). Associations between young adults' use of sexually explicit materials and their sexual preferences, behaviors, and satisfaction. J Sex Res.

[5] Wright PJ, Bae S, Funk M (2013). United States women and pornography through four decades: exposure, attitudes, behaviors, individual differences. Arch Sex Behav.

[6] Leonhardt ND, Spencer TJ, Butler MH, Theobald AC (2019). An organizational framework for sexual Media’s influence on short-term versus long-term sexual quality. Arch Sex Behav.

[7] Wright PJ (2011). Mass media effects on youth sexual behavior assessing the claim for causality. Ann Int Commun Assoc.

[8] Dwulit AD, Rzymski P (2019). The potential associations of pornography use with sexual dysfunctions: an integrative literature review of observational studies. J Clin Med.

[9] Grubbs JB, Gola M (2019). Is pornography use related to erectile functioning? Results from cross-sectional and latent growth curve analyses. J Sex Med.

[10] Shuler J, Brosi M, Spencer T, Hubler D (2021). Pornography and romantic relationships: a qualitative examination of individual experiences. J Sex Marital Ther.

[11] Kohut T, Fisher WA, Campbell L (2017). Perceived effects of pornography on the couple relationship: initial findings of open-ended, participant-informed, "bottom-up" research. Arch Sex Behav.

[12] Campbell L, Kohut T (2017). The use and effects of pornography in romantic relationships. Curr Opin Psychol.

[13] Braithwaite SR, Coulson G, Keddington K, Fincham FD (2015). The influence of pornography on sexual scripts and hooking up among emerging adults in college. Arch Sex Behav.

[14] Martyniuk U, Okolski L, Dekker A (2019). Pornographic content and real-life sexual experiences: findings from a survey of German University students. J Sex Marital Ther.

[15] Fisher WA, Kohut T (2020). Reading pornography: methodological considerations in evaluating pornography research. J Sex Med.

[16] Bothe B, Toth-Kiraly I, Bella N, Potenza MN, Demetrovics Z, Orosz G (2020). Why do people watch pornography? The motivational basis of pornography use. Psychol Addict Behav.

[17] Doring N, Daneback K, Shaughnessy K, Grov C, Byers ES (2017). Online sexual activity experiences among college students: a four-country comparison. Arch Sex Behav.

[18] Hald GM (2006). Gender differences in pornography consumption among young heterosexual Danish adults. Arch Sex Behav.

[19] Martyniuk U, Briken P, Sehner S, Richter-Appelt H, Dekker A (2016). Pornography use and sexual behavior among polish and German University students. J Sex Marital Ther.

[20] Hald GM, Štulhofer A (2016). What types of pornography do people use and do they cluster? Assessing types and categories of pornography consumption in a large-scale online sample. J Sex Res.

[21] Koletić G, Štulhofer A, Hald GM, Træen B (2021). Self-assessed effects of pornography use on personal sex life: results from a large-scale study of Norwegian adults. Int J Sex Health.

[22] Castro Á, Barrada JR, Ramos-Villagrasa PJ, Fernández-Del-Río E (2020). Profiling dating apps users: sociodemographic and personality characteristics. Int J Environ Res Public Health.

[23] Wright PJ, Sun C, Steffen N (2018). Pornography consumption, perceptions of pornography as sexual information, and condom use. J Sex Marital Ther..

[24] Milas G, Wright P, Štulhofer A (2020). Longitudinal assessment of the association between pornography use and sexual satisfaction in adolescence. J Sex Res..

[25] McNabney SM, Heves K, Rowland DL (2020). Effects of pornography use and demographic parameters on sexual response during masturbation and partnered sex in women. Int J Env Res Pub He.

[26] Harkness EL, Mullan B, Blaszczynski A (2015). Association between pornography use and sexual risk behaviors in adult consumers: a systematic review. Cyberpsychol Behav Soc Netw.

[27] Sinković M, Stulhofer A, Božić J (2013). Revisiting the association between pornography use and risky sexual behaviors: the role of early exposure to pornography and sexual sensation seeking. J Sex Res..

[28] McKee A, Litsou K, Byron P, Ingham R (2021). The relationship between consumption of pornography and consensual sexual practice: results of a mixed method systematic review. Can J Hum Sex.

[29] Diamantopoulos A, Sarstedt M, Fuchs M, Wilczynski P, Kaiser S (2012). Guidelines for choosing between multi-item and single-item scales for construct measurement: a predictive validity perspective. J Acad Mark Sci.

[30] Cheung F, Lucas RE (2014). Assessing the validity of single-item life satisfaction measures: results from three large samples. Qual Life Res.

